# Building Technological Legitimacy: The Impact of Communication Strategies on Public Acceptance of Genetically Modified Foods in China

**DOI:** 10.3390/foods14223827

**Published:** 2025-11-08

**Authors:** Yijing Xin, Jiping Sheng

**Affiliations:** School of Agricultural Economics and Rural Development, Renmin University, Beijing 100872, China; xinyijing@ruc.edu.cn

**Keywords:** genetically modified foods, consumer attitudes, communication strategy, China, technological legitimacy

## Abstract

Public acceptance remains a critical barrier to the adoption of genetically modified (GM) foods. This study investigates whether communication strategies that establish different forms of technological legitimacy, specifically regulative, normative, and cognitive legitimacy, can effectively overcome this barrier. Using the contingent valuation method (CVM) with a nationally representative sample of 1194 individuals, this study examined the effect of communication strategies on Chinese consumers’ willingness to pay for GM soybean oil. The results revealed a striking asymmetry. Information emphasizing the safety regulations of GM foods, which aims to build regulative legitimacy, significantly reduced WTP, likely by activating consumer anxieties. Conversely, narratives highlighting technology’s role in ensuring national food security, which builds normative legitimacy, effectively increased WTP for domestic GM oil. Information about the advanced level of GM technology, intended to establish cognitive legitimacy, had no significant impact. The effects were heterogeneous. Females and less knowledgeable consumers were most sensitive to safety messages. Our findings demonstrate that building legitimacy through normative appeals to collective welfare is more effective than relying on regulatory assurances. This study provides a legitimacy-based framework for understanding public perception and offers policymakers crucial insights for communicating about controversial agricultural technologies.

## 1. Introduction

The global food system faces the dual challenges of ensuring food security for a growing population while meeting demands for safe and high-quality nutrition [1,2]. Genetically modified (GM) crops are central to this debate, as they offer a potential solution to enhance yields and agricultural resilience [3,4,5,6,7]. However, their adoption is often hindered by persistent public skepticism regarding safety and potential long-term health impacts [8,9]. Within this context, public communication plays a decisive role in shaping how people perceive GM foods [10,11,12]. It is important to know which types of messages most effectively influence consumer attitudes and purchasing decisions today [13,14,15].

As one of the world’s largest grain consumer, China is a critical case for understanding these dynamics. The country relies heavily on soybean imports, with imports reaching 86.18 million tons in the first three quarters of 2025 alone [16]. In response, the government has accelerated the commercialization of GM soybean and corn in 2023 [17], with a projected planting area of 31 million mu in 2025 [18]. This milestone reflects both the maturity of the technology and a strategic response to international pressure. However, consumer attitude remains uncertain. Early public discussions about GM technology in China have created a complex landscape of public understanding [10,19]. This gap between state-led legitimization and public perception constitutes the central problem that strategic communication must address.

Bridging this acceptance gap is fundamentally a challenge of technological legitimacy, the process by which a technology gains social license and is perceived as desirable, proper, and appropriate within a social system [20,21]. According to this framework, legitimacy is built upon three distinct pillars: regulative (compliance with rules and safety regulations), normative (alignment with moral values and social purposes), and cognitive (technical maturity and its comprehensibility) [22]. Public communication is a primary mechanism for constructing these pillars [23]. Yet, although the importance of communication is acknowledged, a critical unanswered question remains: Which specific pathway to legitimacy is most effective in persuading a skeptical public? The existing literature has often examined information types in isolation, lacking a unified theoretical framework to systematically compare the efficacy of strategies targeting these distinct pillars of legitimacy [23].

Understanding which strategy is most effective is not just a theoretical exercise. It has significant practical consequences. The consumer preferences for domestic GM oil, imported GM oil, and non-GM oil revealed in this study directly impact China’s agricultural and trade policies. If consumers strongly prefer imported or non-GM oil, China will continue to rely on foreign markets [24,25]. This would undermine the stated goals of food security and technological self-sufficiency that underpin the domestic GM commercialization policy [26]. Conversely, acceptance of domestic GM oil would validate the national strategy and provide the social license necessary for the industry to reach its economic and strategic potential [27].

This study examines how different types of communication strategies influence consumer attitudes toward GM soybean oil to address this challenge. Employing an experimental design, we compare three communication strategies, each designed to build a distinct pillar of technological legitimacy: a norm-oriented strategy (emphasizing contribution to national food security), a cognition-oriented strategy (highlighting technological reliability and maturity), and a regulative-oriented strategy (focusing on regulatory oversight and safety). Then, we assess how these messages affect consumers’ willingness to pay for four categories of soybean oil: unknown-origin GM, domestically produced GM, imported GM, and non-GM. This approach allows us to determine not only which legitimacy-building pathway is most effective overall, but also how its effectiveness varies with product origin.

Our study makes three contributions. First, it theorizes and empirically tests the concept of technological legitimacy in the context of GM food acceptance in China, providing a unified framework to compare the efficacy of regulative, normative, and cognitive communication strategies. Second, it introduces a critical empirical distinction between consumer preferences for domestic versus imported GM products, moving beyond a monolithic view of GM acceptance. Third, it reveals a counter-intuitive finding that safety assurances can backfire, offering crucial insights for developing more effective, context-sensitive science communication and policy.

## 2. Literature Review

### 2.1. Theoretical Framework: Technological Legitimacy

Securing public acceptance of novel technologies depends on the process of technological legitimation, through which a technology gains a social license and becomes perceived as desirable, proper, and appropriate within its societal context [21,23,28]. This process, grounded in institutional theory, is governed by the institutional environment, which comprises a complex web of formal and informal social rules and norms [29]. Scott provides a seminal framework for understanding this environment [22]. He argues that legitimacy is built upon three pillars: the regulative pillar, which involves establishing legitimacy through conformity to formal rules, laws, and safety certifications; the normative pillar, which derives from aligning the technology with prevailing moral values, social responsibilities, and conceptions of the public good; and the cognitive pillar, which rests on the comprehensibility of the technology and its status as a mature, reliable solution [22,30]. While these pillars are distinct, they often coexist and interact, and are crucial for securing widespread societal acceptance [31].

The three pillars of legitimacy offer a powerful perspective through which to interpret the divergent global reception of GM crops, reflecting distinct institutional assumptions. For example, a study on GM food in the Netherlands found that the public debate was predominantly negative and centered on the normative pillar, such as ethics and naturalness. Emotional rhetoric overshadowed the cognitive pillar, which includes knowledge and understanding [23]. This pattern aligns with the European Union’s broader approach, which operates under the precautionary principle [32]. The EU treats GM technology as novel and potentially risky, thereby erecting strict regulatory barriers. In contrast, United States policy is based on the idea of substantial equivalence with conventional breeding [6]. It prioritizes a regulatory pathway led by agency approvals, such as those from the FDA, and is supported by a cognitive narrative of scientific consensus [33]. In China, initially, in response to significant public apprehension about safety, efforts to build regulative legitimacy centered on communicating the existence of stringent governmental oversight and scientific risk assessments [19,34]. More recently, against a backdrop of trade tensions and national strategic priorities [26], a powerful narrative has emerged to forge normative legitimacy.

### 2.2. GM Foods Consumption and Consumer Preferences

Soybean oil plays a pivotal role in China’s food system and national food security strategy. As the world’s largest soybean importer, China relies heavily on foreign supplies, creating a strategic vulnerability in its edible oil supply [24,35]. This high level of import dependency fundamentally shapes the policy landscape and underscores the importance of developing domestic agricultural technologies, such as GM crops, to enhance self-sufficiency [36]. Understanding consumer acceptance of GM soybean oil is critically important within this specific market and policy context.

Willingness to pay serves as a key metric for gauging consumer acceptance of GM foods, and it is profoundly influenced by the information environment. Research in GM food communication consistently shows that the type of informational content provided has distinct effects. risk-oriented messages often cite concerns from environmental groups [37,38], while benefit-oriented messages typically cite evidence from research institutions or government bodies [39,40]. The impact of these different types of content on consumer acceptance is inconclusive due to varying findings caused by contextual and methodological factors. While negative information consistently reduces acceptance, the effectiveness of positive information is more uncertain [15,41,42,43]. This uncertainty highlights the importance of identifying effective positive messages for GM risk communication. This asymmetry underscores the critical importance of identifying which specific types of positive information can effectively shape consumer valuation.

Beyond information, the origin of a technology or product is a well-established factor in consumer evaluation, known as the “country-of-origin” effect [44,45,46]. In the context of emerging technologies and international trade tensions, this can manifest as “techno-nationalism,” where consumers exhibit a preference for domestically developed alternatives [44,47,48]. This effect occurs when consumers perceive the quality, safety, and value of a product based on its geographic origin. Thus, it influences their decision-making. For GM soybean oil in China, this implies that consumer WTP may differ significantly between domestically produced, imported, and unknown origin products. However, studies that systematically examine how the effectiveness of different information strategies varies across GM product origins are scarce [27]. This creates a gap in our understanding of how to communicate effectively about GM foods within a unified theoretical framework that considers both informational content and product origin.

## 3. Materials and Methods

### 3.1. Survey Design

To address this gap and deliver on our stated contributions, we designed a randomized controlled trial grounded in the theory of technological legitimacy. This study identified two key factors. The first is the communication strategy, consisting of three distinct strategies targeting regulatory, normative, and cognitive legitimacy. The second factor is product origin, including domestically produced soybean oil, imported soybean oil, and soybean oil of unknown origin. This design enables us to theorize and empirically test the concept of technological legitimacy in the Chinese context by comparing the efficacy of these strategies. Second, we can measure how these strategies influence consumer preferences for domestic versus imported products. Finally, we can explore heterogeneity in information effects across consumer groups to derive implications for more targeted communication strategies. We conducted the experiment in China, the world’s largest soybean importer, where these issues have significant policy implications.

In a randomized controlled design, participants were randomly assigned to one of four treatment groups. The first group received no information and served as the control group (*n* = 295). The experimental treatments were designed to operationalize the pillars of technological legitimacy theory (Table 1). The second group was exposed to a purpose-oriented strategy that aimed to build normative legitimacy by emphasizing the public benefits of domestically produced GM soybeans and their role in addressing food sufficiency challenges (*n* = 304). The third group received a maturity-oriented strategy designed to establish cognitive legitimacy by emphasizing that China’s GM soybean technology is mature and has its own intellectual property rights (*n* = 296). The fourth group received a safety-oriented strategy intended to foster regulatory legitimacy by emphasizing the rigorous safety evaluation and approval procedures that all Chinese GM crops undergo (*n* = 300). To ensure credibility, all messages were framed as originating from the Special Topic on Genetically Modified Food website of the Chinese Ministry of Agriculture and Rural Affairs (“Transgenic Authority Focus” of the Ministry of Agriculture and Rural Affairs of China: https://www.moa.gov.cn/ztzl/zjyqwgz/, accessed on 1 October 2023) [49].

The survey was conducted online in China in October 2023 using WenjuanXing, a widely used mobile survey platform with more than one billion active users nationwide. Using this platform allowed us to access a diverse pool of respondents from across different regions. Participants were randomly assigned to one of four experimental groups using a computer-generated randomization algorithm administered by the platform. Participants were assigned to a group immediately after qualifying for this study, before any informational stimuli or survey questions were presented. Each respondent completed the questionnaire once. Screening questions ensured the quality of the responses, all respondents were aged 18 or older and had purchased soybean oil. All participants provided informed consent, and ethical standards were strictly adhered to. Their responses were anonymized to ensure confidentiality.

The survey was conducted in five stages. First, respondents provided information about their demographics, including their gender, level of education, income, and age. Second, they answered questions about their knowledge of biotechnology and GM technology, their consumption habits regarding GM foods, their perceptions of the safety of GM foods, and their acceptance levels toward different types of GM foods. Third, they reported their sources of GM-related information, their level of trust in these sources, their need for additional knowledge, and their awareness of and preferences regarding GM labeling and compositional improvements. Fourth, participants were exposed to an informational intervention about GM foods. Finally, they indicated their WTP for specific GM food products.

### 3.2. Contingent Valuation Experiment

**Figure 1:**
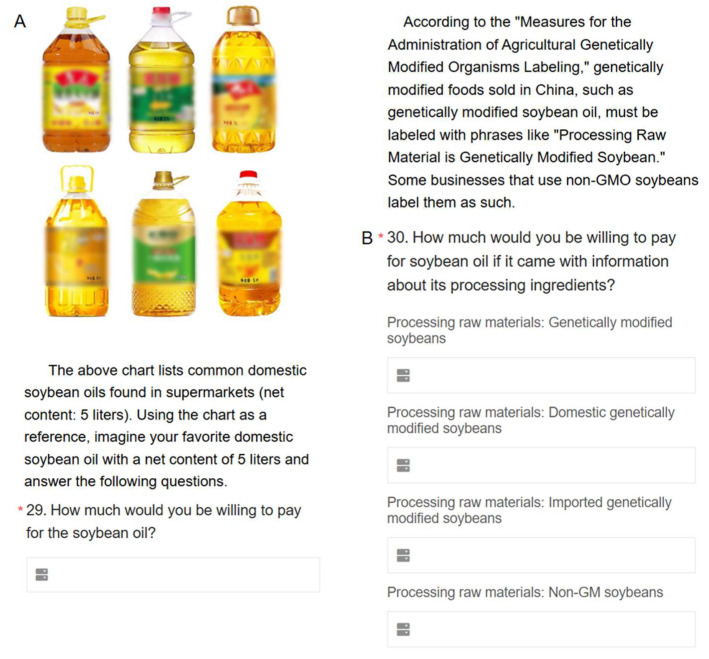
Pictures of the type of soybean oil consumers usually buy in grocery stores (**A**). Four soybean oil label examples (**B**). * It comes with the questionnaire and means that this question must be answered.

The contingent valuation method (CVM) using payment cards was employed to investigate consumers’ WTP for soybean oil with different raw material labels (Figure 1). This method was chosen because it effectively addresses issues like zero observations and starting point bias, which are often encountered in other CVM approaches [50].

In this study, the hypothetical product was five liters of soybean oil, which is the most common package size for household consumption in China. First, respondents were first asked to indicate the maximum price they would pay for soybean oil with no specific labeling. Considering that the average market price of domestically produced soybean oil in October 2023 was obtained from physical supermarkets as well as online platforms such as JD.com and Taobao, the price interval was set from 0 to 100 yuan. The payment card design initially used an interval of 10 yuan, followed by narrower 1-yuan intervals based on respondents’ initial choices. This allowed for a more precise measurement of WTP.

After eliciting the baseline WTP, respondents were further asked to state their WTP for soybean oil with different labels. According to the Regulations on the Administration of Agricultural Genetically Modified Organism Labeling in China, GM soybean oil products must carry a mandatory label indicating that they are “produced from genetically modified soybeans.” Meanwhile, some producers voluntarily label their products as “non-GMO” to distinguish them in the marketplace when using non-GM soybeans. To capture consumer preferences across different raw material claims, the experiment included four label categories: (1) GMO soybean oil, (2) domestically produced GMO soybean oil, (3) imported GMO soybean oil, and (4) non-GMO soybean oil. Respondents were asked to select their WTP for each category using the payment card procedure.

### 3.3. Data and Variable Description

A total of 1195 respondents completed the survey. They were required to be at least 18 years old and were recruited from a nationally representative sample covering 29 provinces in China. Table 1 provides summary statistics on the characteristics of the sample.

The sample is nearly balanced in terms of gender, with females accounting for 56.37%. The age distribution shows that most respondents are between 26 and 40 years old (72.28%), constituting the core working-age population. In terms of educational attainment, the majority (85.18%) hold a college or bachelor’s degree, while 10.13% possess a master’s degree or higher. The monthly income distribution is relatively even across the predefined categories. The average biotechnology knowledge score is 3.77 on a 5-point scale.

As shown in Table 2, the statistical tests confirm that the randomization procedure was successful. Key demographic variables, including age, gender, income, education level, occupation, and biotech knowledge, are balanced between the control and treatment groups. No statistically significant differences were observed. This balance ensures that any subsequent observed differences between groups can be more confidently attributed to the experimental treatments rather than to pre-existing differences in sample composition.

### 3.4. Econometric Model

The WTP were elicited using a payment card design. With this approach, respondents indicate their maximum willingness to pay selecting from a set of predetermined price ranges. This generates an interval rather than a point estimate of WTP for each observation. Let the unobserved latent WTP of individual *i* for product *j* be denoted by ${WTP}_{ij}^{*}$. The survey response implies that:

$$L_{ij}\leq {WTP}_{ij}^{*}\leq U_{ij},$$

where $L_{ij}$ and $U_{ij}$ represent the lower and upper bounds of the payment card interval selected by the respondent. Observations selecting the lowest or highest categories are treated as left-censored or right-censored, respectively. This data structure is appropriately modeled using an interval regression. The likelihood contribution for each observation is the probability that the latent WTP falls within the reported interval:

$$Pr\left(L_{ij}\leq {WTP}_{ij}^{*}\leq U_{ij}\right),$$

which is evaluated under the assumption of normally distributed errors. Estimation proceeds by maximizing the log-likelihood function for all respondents.

The econometric specification of the latent WTP is given by:

$${WTP}_{ij}^{*}\;=\;\beta_{0}\;+\;\beta_{1}{Purpose}_{i}\;+\;\beta_{2}{Maturity}_{i}\;+\;\beta_{3}{Safety}_{i}\;+\;\gamma X_{i}\;+\;\varepsilon_{ij},$$

where ${Purpose}_{i}$, ${Maturity}_{i}$, ${Safety}_{i}$ are dummy variables indicating the assignment to the respective information treatment groups, with the control group omitted as the baseline. $X_{i}$ is a vector of control variables, including demographic characteristics and consumption habits; and $\varepsilon_{ij}$ is the error term.

## 4. Results

### 4.1. Statistics of WTP

This section reports the estimated mean WTP for soybean oil under different information treatments, as shown in Table 3. All average WTP estimates are statistically different from zero at the 1% level. The most consistent finding is that across all experimental groups, consumers demonstrate a higher WTP for non-GMO soybean oil than for any of the three GM types.

Within-group comparisons confirm this pattern. Kruskal–Wallis tests consistently showed that the WTP for non-GMO soybean oil was significantly higher than that for all GM alternatives, including the no-information control group and all three information treatment groups (purpose, maturity, and safety), at the 1% significance level.

Between-group comparisons, tested using Kruskal–Wallis tests, provide insight into the impact of information. For generic GM soybean oil, the WTP in the safety strategy group is significantly lower than in the other three groups at the 1% significance level. For Domestic GM soybean oil, the WTP in the safety strategy group is significantly lower than in all other groups at the 1% level. Meanwhile, the WTP in the purpose strategy group is significantly higher than in the control group at the 5% level. In addition, the safety strategy also significantly reduced WTP for imported GM soybean oil compared to the other groups at the 1% level. In stark contrast to the GM products, there are no statistically significant differences in WTP for non-GMO soybean oil across any of the information treatment groups.

### 4.2. Information Interventions and Determinants of WTP

This section examines the impact of different information interventions, demographics, knowledge, and perceptions of GM foods on consumers’ WTP for the four types of soybean oil. This examination is based on the regression results presented in Table 4.

The findings suggest that information strategies significantly impact WTP, though the effects vary across products. The purpose strategy had a positive and statistically significant effect on WTP for domestic GM soybean oil, increasing it by 3.308 yuan compared to the no-information baseline. In contrast, it has no significant impact on generic GM, imported GM, or non-GMO soybean oil. Similarly, the Maturity strategy leads to modest increases in WTP across all GM types, though these effects are not statistically significant. It appears to have a negligible influence on non-GMO products. The most pronounced effects are observed under the Safety strategy, which significantly decreases WTP for all four products. The largest decrease is seen for generic GM soybean oil, with a decline of 5.600 yuan. Imported GM and domestic GM soybean oils decrease by 5.205 and 5.053 yuan, respectively. Even non-GMO soybean oil experiences a significant reduction in WTP of 4.012 yuan under this strategy. These results suggest that safety strategy exerts a strong negative influence on consumers’ valuation of soybean oil, regardless of its genetic modification status. Conversely, purpose strategy can increase WTP for domestic GM products specifically, highlighting the potential of targeted messaging to influence consumer preferences.

The regression results further clarify the effects of demographic characteristics and consumer perceptions on WTP. Age has a significant negative effect on WTP for all three types of GM soybean oil, which is significant at the 1% level. Compared to government employees, students, and individuals those working in enterprises, public institutions, and agricultural production, show significantly lower WTP for generic GM soybean oil. This difference is significant at the 5% level.

Socioeconomic factors display a distinct pattern. Higher education levels and income are not significantly associated with WTP for any GM product. However, they are positively correlated with the WTP for non-GMO oil. Holding a university degree is significant at the 10% level, while a master’s degree or higher and membership in higher income brackets are significant at the 5% and 1% levels, respectively. These results suggest that individuals with higher socioeconomic status place a greater premium on non-GM attributes.

Consumer knowledge and perceptions are strong determinants. Higher self-reported knowledge of biotechnology is positively associated with WTP for generic GM oil, which is significant at the 5% level. The perception of GM food safety is an exceptionally powerful driver, showing a positive and highly significant association with WTP for all three GM products at the 1% level. This safety perception has no significant effect on the WTP for non-GMO oil. Finally, respondents who pay greater attention to soybean oil prices report a consistently higher WTP across all categories, significant at the 1% level. This likely reflects their role as primary purchasers.

### 4.3. Heterogeneity Analysis

This section examines how information interventions affect different consumer subgroups. All estimated coefficients represent the change in WTP relative to the baseline no-information baseline group. The results are shown in Table 5.

The analysis of demographic heterogeneities reveals striking patterns. Gender emerges as a significant moderator of treatment effects. The safety strategy triggered significant WTP reductions among females across all oil types, with significant decreases for generic GM at the 1% level, and for domestic GM, imported GM, and non-GMO significant at the 1%, 1%, and 5% levels, respectively. Conversely, male consumers exhibited no significant response to negative safety information. Instead, their WTP for domestic GM oil increased significantly at the 10% level under both purpose and maturity strategies. The presence of children similarly differentiated the responses. Parents reduced their WTP for all three GM types when exposed to the safety strategy, reaching significant at the 5% or 10% levels, while childless households increased their WTP for domestic GM oil at the 10% significance level following the same intervention.

Cognitive and perceptual heterogeneities further shape treatment efficacy. The manner in which consumers processed information was determined by their respective knowledge levels. The maturity strategy significantly raised WTP for generic and domestic GM oil among high-knowledge consumers at the 10% level. Conversely, the safety strategy had a negative impact on low-knowledge consumers, reducing their WTP for all three GM products at the 5% significance level. Furthermore, pre-existing safety perceptions had a similar heterogeneity effect. Consumers with low safety perceptions responded positively to purpose and maturity strategies for domestic GM oil at the 1% and 5% level, respectively. Those with high safety perceptions, however, reacted negatively to the safety strategy, reducing their WTP for generic and domestic GM oil at the 5% and 10% significance levels, respectively.

## 5. Discussion

This study provides experimental evidence regarding the association between information strategies that emphasize safety regulations and consumers’ willingness to pay (WTP) for GM soybean oil in China. The findings reveal nuanced and heterogeneous effects, offering important insights for both theory and practice.

The most significant finding of this study is the substantial correlation between the safety strategy and consumers’ WTP for GM products. The intervention was designed to emphasize positive regulatory oversight and scientific consensus on safety, not to present negative or alarmist information. Nevertheless, it corresponded to a significant and remarkably consistent decrease in WTP across all three GM types. This result suggests that the mere mention of the topic of safety, even in a reassuring, regulatory context, can activate pre-existing consumer anxieties and skepticism, a form of policy backlash or reactance. This finding aligns with consumer reactance phenomenon [51,52]. It indicates that attempts to reassure can sometimes paradoxically amplify risk perceptions by making the risk itself more salient. The fact that this strategy was also associated with lower WTP for non-GMO oil, though to a lesser extent, further underscores its pervasive effect of raising generalized safety concerns across the entire product category.

In contrast, the purpose strategy positively influenced WTP for domestic GM oil, particularly among male consumers and those with initially low safety perceptions. This highlights the effectiveness of benefit-oriented strategy, especially when combined with a domestic source. These results suggest that consumers may be more receptive to GM technologies when their tangible benefits are clearly communicated and the products originate from a trusted domestic system. The maturity strategy had modest positive effects, primarily among consumers with higher knowledge levels. This indicates that technical proficiency information may only resonate with a subset of the population that is more informed.

The heterogeneity analysis revealed that these treatment effects were not uniform but instead were powerfully moderated by consumer demographics and cognitions. The pronounced gender gap, in which women accounted for all of the negative effects of the safety strategy, is consistent with the literature showing that women often exhibit greater risk sensitivity and often act as primary household food safety gatekeepers [53,54]. Similarly, parents’ heightened responsiveness to the safety message underscores the influence on food choices, as decisions are often made to protect children [55,56].

Another critical moderating factor is prior knowledge. The negative association with the safety strategy was most pronounced among consumers with low knowledge, resulting in a significant decrease in their WTP. This indicates that, for this group, any mention of safety may cause confusion and distrust rather than confidence. This highlights the limitations of the knowledge deficit model of communication [57,58]. High-knowledge consumers, who were able to contextualize the regulatory information, were not significantly affected. This underscores the importance of a baseline level of public understanding for effective science communication.

This study reveals a critical tension in the legitimization of GM technology in China. Although the current policy framework emphasizes scientific oversight and regulatory rigor [49], our experimental evidence suggests that public communication focused on establishing regulatory legitimacy through safety assurances could be counterproductive. Public acceptance is more effectively built through narratives that establish normative legitimacy by integrating the technology into national strategic initiatives, particularly those related to food security. This finding has direct implications for implementing China’s commercialization policy, suggesting that communication strategies should prioritize normative alignment over regulatory reassurance. In a cross-cultural context, this pattern highlights China’s uniqueness. Unlike the United States, where legitimation often follows a regulatory pathway focused on agency approvals, and unlike the European Union, where public debate is dominated by normative concerns over naturalness [6,33], China’s model demonstrates the importance of state-aligned normative framing in the public sphere. Consequently, converting policy momentum into genuine public acceptance may depend less on emphasizing regulatory rigor or technical maturity and more on strategically integrating GM technology into the prevailing normative fabric of collective progress and security.

## 6. Conclusions

This study shows that the effectiveness of information strategies in shaping public acceptance of GM technology depends heavily on message content and audience characteristics. Although well-intentioned, emphasizing safety regulations can be a double-edged sword, potentially exacerbating public concerns instead of alleviating them.

These findings have important implications for stakeholders involved in China’s GM commercialization efforts. For policymakers, our results strongly suggest shifting the strategic focus of public communication from safety reassurance to articulating tangible benefits. This shift should emphasize narratives about national food security and technological self-sufficiency, core tenets of China’s current GM policy framework. For industry stakeholders, the positive response to purpose-driven messages for domestic products reveals a clear competitive advantage for Domestic GM that should be leveraged through marketing and branding that emphasizes contributions to China’s food sovereignty. For science communicators, the limited resonance of technical maturity information and the pronounced reactance to safety messages among less knowledgeable consumers highlight the necessity of moving beyond the simplistic knowledge deficit model. This requires building foundational biotechnology literacy through value-based narratives. In sum, achieving societal acceptance of GM technology in China requires replacing generic assurances with sophisticated communication strategies that are as advanced as the technology they promote and that are sensitive to audience needs.

This study has several important limitations. First, the hypothetical nature of our WTP measure and the online sampling method may affect the external validity of absolute value estimates. However, the randomized design ensures the robustness of the relative treatment effects. Second, since this study was conducted within China’s specific sociocultural context, the generalizability of the findings, particularly the strong reactance to safety assurances, to other cultural settings remains to be tested. Third, although our national sample captured geographic diversity, we did not systematically analyze regional heterogeneity or urban-rural differences that could affect strategy effectiveness. Finally, self-reported data from a single survey may be subject to common method bias, and multiple responses per participant create the potential for autocorrelation. Future research would benefit from cross-cultural validation, real-market behavioral studies, regionally stratified designs, and methods that account for within-subject dependencies to further refine these communication strategies.

## Figures and Tables

**Table 1:** Messages presented in treatment groups.

Information Group	Message
Purpose strategy	Soybeans are an important raw material for edible oils, soy products, and animal feed in our country, and the demand for them has grown rapidly in recent years. However, due to the low yield of conventional soybeans and the limited amount of arable land per capita, it is difficult for China to achieve self-sufficiency in soybean production. To rely solely on conventional soybeans for self-sufficiency, China would need to increase its soybean area by an additional 800 million mu, which is equivalent to dedicating 45% of its current arable land to soybean cultivation. As a result, we have long relied on soybean imports to meet our needs, importing 91.08 million tons in 2022 from countries such as Brazil and the United States, mostly GM soybeans. This has led to external soybean dependence of more than 80%, making us vulnerable to other countries in terms of food security. Given the complex and volatile international situation, there is an urgent need to accelerate the industrialization of GM soybean breeding. Using independently developed GM technology to increase soybean self-sufficiency, we can ensure food security.
Maturity strategy	GM technology is an important means to enhance the competitiveness of soybean industry in China. After more than 20 years of scientific and technological innovation, breeding techniques for herbicide tolerance and insect resistance in GM soybeans have matured. GM varieties that have been approved for pilot testing now have independent intellectual property rights in China, and the industrialized cultivation of these products can significantly reduce production costs and increase soybean yields. Taking herbicide-resistant transgenic soybeans as an example, as of June 2023, five herbicide-resistant soybean varieties bred using transgenic technology had received safety certificates for production and use. These varieties can reduce weed control costs by more than 30 RMB per mu, increase yields by over 10% compared to main crop varieties, and improve efficiency by an average of 100 RMB per mu while enabling reasonable crop rotation. Our proprietary herbicide-tolerant soybean has also been approved for commercial cultivation in Argentina, completing the international rollout of transgenic products.
Safety strategy	In China, all GM crops go through a series of rigorous safety evaluations and approvals before being released to the market. The process is based on internationally accepted practices, as well as national laws, regulations, and standard specifications. The process is divided into five stages, including experimental research, intermediate testing, environmental release, production testing and application for safety certificates. It includes the safety evaluation of both food safety risks and environmental safety risks. If any problems that may affect health or environmental safety are found at any stage, the R&D testing stops immediately and does not proceed to the industrialization stage. For example, the domestic GM soybean, Zhonghuang 6106, received a safety certificate for production and use. This certification followed an extensive, 11-year evaluation by the National Committee on the Safety of Genetically Modified Organisms in Agriculture. The committee systematically assessed the soybean’s safety for human consumption and its impact on the environment.

**Table 2:** Frequency distribution of respondents’ characteristics.

Variable	Description	Percentage				
		Total	Control	Treatment1	Treatment2	Treatment3
Gender	Female	56.37	60.34	52.15	54.73	58.33
	Male	43.63	39.66	47.85	45.27	41.67
Age	18–25	14.99	13.90	14.52	15.54	16.00
	26–30	29.31	27.80	31.68	31.42	26.33
	31–40	42.97	43.73	43.23	40.20	44.67
	41–50	9.46	11.53	6.60	9.12	10.67
	51–60	3.10	2.71	3.96	3.38	2.33
	Above 60	0.17	0.34	0.00	0.34	0.00
Education	High school and below	4.69	3.39	4.62	6.42	4.33
	Some college and Bachelor’s degree	85.18	89.83	82.18	83.45	85.33
	Master’s degree or equivalent	10.13	6.78	13.20	10.14	10.33
Monthly Income	Under 4999 yuan	4.77	4.75	5.28	5.74	3.33
	5000–9999 yuan	20.69	18.31	21.45	20.95	22.00
	10,000–14,999 yuan	25.96	29.15	24.75	24.66	25.33
	15,000–19,999 yuan	23.79	19.32	28.05	22.97	24.67
	20,000 yuan and above	24.79	28.47	20.46	25.68	24.67
Knowledge	Mean value	3.77	3.69	3.91	3.73	3.74

**Table 3:** Estimated average WTPs by information treatment.

	GM	Domestic GM	Imported GM	Non-GMO
No information	45.482	46.564	46.426	62.328
	(1.391)	(1.398)	(1.441)	(1.428)
Purpose strategy	45.881	49.872	46.747	62.139
	(1.372)	(1.379)	(1.422)	(1.409)
Maturity strategy	47.753	49.039	47.994	61.867
	(1.382)	(1.389)	(1.433)	(1.419)
Safety strategy	39.882	41.511	41.221	58.316
	(1.378)	(1.385)	(1.428)	(1.415)

Standard errors in parentheses.

**Table 4:** Results of consumers’ WTP for soybean oil of different information groups.

	(1)	(2)	(3)	(4)
Variables	GM	Domestic GM	Imported GM	Non-GMO
Purpose strategy	0.399	3.308 *	0.321	−0.189
	(1.965)	(1.976)	(2.037)	(2.018)
Maturity strategy	2.271	2.476	1.568	−0.461
	(1.961)	(1.971)	(2.033)	(2.014)
Safety strategy	−5.600 ***	−5.053 **	−5.205 **	−4.012 **
	(1.962)	(1.972)	(2.034)	(2.015)
Gender	−1.527	−1.932	−2.941 **	−2.046
	(1.410)	(1.417)	(1.461)	(1.448)
Age	−0.288 ***	−0.348 ***	−0.326 ***	−0.044
	(0.107)	(0.107)	(0.111)	(0.110)
*Based on government agencies*				
Public Institution	−11.502 **	−8.878	−7.062	−6.017
	(5.416)	(5.444)	(5.613)	(5.561)
Enterprise	−12.018 **	−9.513 *	−7.334	−5.665
	(5.144)	(5.171)	(5.331)	(5.282)
Student	−13.710 **	−9.330	−9.642	−8.324
	(5.959)	(5.990)	(6.176)	(6.119)
Agricultural producers	−20.641 **	−19.338 **	−12.875	−11.671
	(8.590)	(8.634)	(8.902)	(8.820)
Others	−9.950 *	−6.127	−5.162	−7.815
	(5.899)	(5.930)	(6.114)	(6.057)
*Based on the level of high school education or below*				
University or junior college	2.321	1.203	2.969	6.063 *
	(3.413)	(3.430)	(3.537)	(3.504)
Master’s degree or above	2.917	3.334	4.476	9.104 **
	(4.031)	(4.052)	(4.178)	(4.139)
*Based on the low-income group*				
Middle and low income	2.687	1.989	4.057	6.612 *
	(3.578)	(3.597)	(3.708)	(3.674)
Middle income	4.150	2.684	4.798	6.833 *
	(3.589)	(3.608)	(3.720)	(3.685)
Middle and high income levels	3.721	2.387	3.895	9.094 **
	(3.655)	(3.673)	(3.788)	(3.752)
High income	4.268	2.337	4.449	11.691 ***
	(3.674)	(3.693)	(3.807)	(3.772)
people aged 60 and above	−0.754	0.276	−0.001	−1.123
	(1.411)	(1.418)	(1.463)	(1.449)
children under the age of 18	2.840 *	3.644 **	3.322 *	3.741 **
	(1.664)	(1.672)	(1.724)	(1.708)
Biotechnology knowledge	1.256 **	0.677	0.299	0.908
	(0.592)	(0.595)	(0.614)	(0.608)
Perception of GM Food Safety	8.651 ***	9.336 ***	9.946 ***	−0.350
	(0.785)	(0.789)	(0.813)	(0.806)
Pay attention to the price of soybean oil	5.126 ***	6.702 ***	7.009 ***	5.031 ***
	(1.848)	(1.858)	(1.915)	(1.898)
Intercept term	23.476 ***	23.790 ***	17.671 *	47.852 ***
	(8.874)	(8.919)	(9.196)	(9.111)
Sample size	1194	1194	1194	1194

Standard errors in parentheses, *** *p* < 0.01, ** *p* < 0.05, * *p* < 0.1.

**Table 5:** Heterogeneous Effects of Information Treatments on WTP.

		GM	Domestic GM	Imported GM	Non-GMO
Gender	Purpose strategy				
	Female	0.476	3.288	−0.169	−0.010
	Male	1.779	5.046 *	2.100	1.088
	Maturity strategy				
	Female	−0.112	−0.238	−0.639	−2.596
	Male	4.729	5.614 *	3.626	2.306
	Safety strategy				
	Female	−9.553 ***	−8.845 ***	−8.826 ***	−6.206 **
	Male	−1.072	−0.434	−0.804	−0.628
Child	Purpose strategy				
	With child	0.501	4.094 *	0.469	−0.197
	Without child	0.739	1.480	0.330	0.838
	Maturity strategy				
	With child	1.113	2.055	0.397	−1.578
	Without child	6.213	4.501	5.331	3.766
	Safety strategy				
	With child	−6.049 ***	−4.441 *	−5.744 *	−3.638
	Without child	−4.352	−6.765 *	−4.261	−4.351
Biotechnology knowledge	Purpose strategy				
	High knowledge	0.362	3.904	1.063	0.844
	Low knowledge	1.276	2.917	−0.196	−0.898
	Maturity strategy				
	High knowledge	4.169 *	4.559 *	3.548	1.747
	Low knowledge	0.079	0.326	−0.540	−2.022
	Safety strategy				
	High knowledge	−3.471	−2.662	−2.872	−2.211
	Low knowledge	−7.718 **	−7.475 **	−7.675 **	−5.292
Perception of the safety of GM technology	Purpose strategy				
	High awareness	−0.406	0.403	−1.927	−1.568
	Low awareness	2.102	4.766 ***	1.281	2.041
	Maturity strategy				
	High awareness	3.046	−2.152 *	−1.143	−3.322
	Low awareness	2.688	2.206 **	0.0144	−0.909
	Safety strategy				
	High awareness	−7.504 **	−2.029 *	−1.791	1.134
	Low awareness	−4.115	1.652 *	1.226	−2.084

*** *p* < 0.01, ** *p* < 0.05, * *p* < 0.1.

## Data Availability

The data presented in this study are available upon request from the corresponding author. The data are not publicly available due to privacy restrictions.
